# Chemoradiation and the Role of Adjuvant Chemotherapy in Lymph Nodal–Metastatic Cervical Cancer

**DOI:** 10.1200/JGO.2017.009852

**Published:** 2017-06-12

**Authors:** Nasir Ali, Azmina Tajdin Valimohammad, Ahmed Nadeem Abbasi, Muhammad Atif Mansha, Asim Hafiz, Bilal Mazhar Qureshi

**Affiliations:** **All authors**: Aga Khan University Hospital, Karachi, Pakistan.

## Abstract

**Purpose:**

To report the long-term outcome in lymph nodal–metastatic cervical squamous cell cancer after chemoradiation followed by adjuvant chemotherapy.

**Patients and Methods:**

Between 2010 and 2013, five patients were diagnosed with advanced cervical cancer with clinically involved para-aortic lymph nodes (ie, International Federation of Gynecology and Obstetrics stage IVB). These patients were treated with concurrent chemoradiation therapy followed by adjuvant chemotherapy. Concurrent chemoradiation consisted of cisplatin given once per week concomitantly with extended-field radiation therapy followed by high-dose-rate brachytherapy. Adjuvant chemotherapy comprised four courses of carboplatin and paclitaxel given every three weeks. The primary outcomes were local and distant failures.

**Results:**

None of the patients had local recurrence or distal failure after a minimum follow-up time of 3 years.

**Conclusion:**

Adjuvant chemotherapy after chemoradiation has a probable role in the management of lymph nodal–metastatic cervical cancer.

## INTRODUCTION

Cervical cancer is an ominous disease when it presents in locally advanced stages. To date, the standard of care for locally advanced disease is pelvic radiotherapy with concurrent cisplatin-based chemotherapy. However, distant failure remains a major problem that leads to compromised overall survival.^1^ To address this problem, a multinational randomized trial was conducted in 2002,^2^ in which patients received two cycles of adjuvant gemcitabine and cisplatin after completion of definitive chemoradiation therapy. This treatment led to a 10% improvement in 3-year progression-free survival. After this, no other trial was conducted to explore improvements in overall survival.

At our university hospital, we treated two patients in 2010, two in 2012, and one in 2013 with four cycles of adjuvant carboplatin (area under the curve [AUC] 5) and paclitaxel (175 mg/m^2^) after completion of chemoradiation therapy. Two of these patients have completed 5 years of follow-up, two have completed 4 years of follow-up, and one patient has completed 3 years of follow-up. We present a short report of five patients who had lymph nodal–metastatic cervical squamous cell carcinoma.

## PATIENTS AND METHODS

### Patient Case 1

A 45-year-old woman presented with locally advanced squamous cell carcinoma of the cervix. The staging positron emission tomography (PET)/computed tomography (CT) scan revealed a 6 × 8 cm metabolically active cervical mass with pelvic and para-aortic lymph nodes that measured more than 1 cm. She received concurrent cisplatin (40 mg/m^2^/week) and extended-field radiation therapy to the pelvis and the para-aortic region after three-dimensional CT-based planning. The pelvis and para-aortic field received a dose of 45 Gy at 1.8 Gy/day, followed by a boost to 50.4 Gy to the para-aortic and pelvic side wall nodes. She then received three high-dose-rate (HDR) brachytherapy fractions, and an 8-Gy dose was prescribed at point A in each fraction. After completion of chemoradiation therapy and brachytherapy, she received four cycles of carboplatin and paclitaxel. She completed the whole treatment in June 2010. The follow-up PET/CT scan revealed metabolic complete response at nodal and primary sites. She is free of any disease at 6 years of follow-up.

### Patient Case 2

In a 43-year-old woman who presented after partial hysterectomy (performed elsewhere), histopathology was consistent with cervical squamous cell carcinoma, and resection margins were reported to be positive. Her postoperative CT scan revealed 4-cm residual disease at the vaginal vault with left parametrial invasion, multiple pelvic nodes, and a 2-cm enhancing left para-aortic node; also, she had a left pelvic kidney. The treatment plan was as follows: radiation therapy to the pelvis and para-aortic field with a concurrent cisplatin dose of 40 mg/m^2^ once per week. Because of previous partial hysterectomy, brachytherapy could not be performed, so she received additional boost dose to the para-aortic nodes to a total dose of 50.4 Gy and to the pelvic mass to a total dose of 63 Gy at 1.8 Gy/day. After completion of chemoradiation therapy, the patient received four cycles of adjuvant carboplatin and paclitaxel until June 2010. At 5 years of follow-up, she is free of any disease on clinical examination and by CT scan performed in March 2015. Her last follow-up visit was on April 27, 2016.

### Patient Case 3

This 48-year-old multiparous woman presented with biopsy-proven squamous cell carcinoma of the cervix. The staging CT scan revealed a large cervical mass as well as pelvic side wall and para-aortic lymphadenopathy. Para-aortic nodes were measured as more than 2 cm in the short axis. The planned treatment was chemoradiation therapy to the pelvis and para-aortic nodes to a total dose of 45 Gy at 1.8 Gy/fraction with cisplatin 40 mg/m^2^ once per week followed by HDR brachytherapy to a total dose of 24 Gy in three fractions to point A. After completion of chemoradiation therapy, the patient received four cycles of carboplatin and paclitaxel chemotherapy until January 2012. She was free of disease at the last follow-up visit on September 9, 2016.

### Patient Case 4

This 54-year-old woman presented with biopsy-proven squamous cell carcinoma of the cervix; on examination under anesthesia, the disease was staged as IIIB. However, on a staging CT scan of the chest, abdomen, and pelvis, there were multiple para-aortic nodes that were more than 2 cm each just below the left renal hilum. The disease ultimately was staged as IVB squamous cell carcinoma. The planned treatment was extended-field radiation therapy with concurrent chemotherapy followed by HDR brachytherapy, then four cycles of chemotherapy with carboplatin and paclitaxel. The patient completed treatment in October 2012. She was in remission at the last follow-up on October 26, 2016.

### Patient Case 5

This 62-year-old multiparous woman presented with cervical biopsy–proven, nonkeratinizing squamous cell carcinoma. Examination under anesthesia revealed a 6 × 6 cm cervical mass fixed to the right pelvic side wall, and a staging CT scan showed a 3.5 × 7 cm nodal mass just superior to aortic bifurcation on the right side. The patient received 45 Gy per 25 fractions of external-beam radiation therapy to the pelvis and para-aortic field with concurrent cisplatin chemotherapy. In last three cycles of chemotherapy, the dose of cisplatin had to be reduced to 30/mg/m^2^ because of grade II hematologic toxicity. This radiation was followed by a boost to the right para-aortic node up to 60 Gy and HDR brachytherapy at a dose of three fractions of 8 Gy each to point A. After chemoradiation therapy, the patient received four cycles of carboplatin and paclitaxel chemotherapy until June 2013. On the PET/CT scan and a clinical examination at the last follow-up in June 2016, she was free of any disease.

### Radiation Therapy Technique

CT simulation was performed with intravenous contrast in all patients. For immobilization and reproducibility of set-up, an alpha cradle vac-lock was used in CT simulation. The superior border of the field was placed at the top of the D12 vertebral body, and the inferior border was 3 cm inferior to the caudal-most extent of the disease in the vagina. A normal saline–soaked gauze piece was put into the vagina for a negative contrast on imaging. A radio-opaque marker was placed at the vaginal introitus, and a plastic tube was placed in the anal canal for facilitation of visualization of organs at risk. Lateral borders were placed 2 cm away from the pelvic bony margin and 1 cm away from the para-aortic volume of the nodes, and multileaf collimators were used to shield normal tissue. Extended pelvis and para-aortic fields were treated each day with antero-posterior (AP), postero-anterior (PA), and right and left lateral fields with 18 MV of photon energy. The radiation planning was performed on Aria 10 planning software (Varian, Palo Alto, CA).

Complete blood counts and serum creatinine were checked once per week. All of the fields were treated each day for 5 days per week. Treatment-verification portal films were taken once per week for accuracy of treatment fields.

In the last week of external-beam radiotherapy, first fraction of HDR brachytherapy was instituted with tandem and ovoid applicators. The brachytherapy procedure was performed under general anesthesia, and careful gauze-piece packing was performed to displace the rectum and bladder away from the applicators. The HDR brachytherapy dose was prescribed at point A according to International Commission on Radiation Units and Measurements report 38.

## RESULTS AND DISCUSSION

Advanced cervical cancer is a virulent disease, and the 5-year disease-free survival rate is dismal. One attempt was made to improve overall survival in locally advanced cervical cancer in a randomized trial that compared adjuvant cisplatin and gemcitabine versus no additional chemotherapy. This trial showed a 10% improvement in the 3-year progression-free survival, but improvement was at the cost of slightly increased toxicity seen in the group that received gemcitabine and cisplatin.^3^ In a retrospective analysis, Kim et al^4^ failed to show a significant difference in survival in patients who received adjuvant chemotherapy in locally advanced cervical cancer. In a phase II trial, Chung et al^5^ treated patients with extended-field radiotherapy, concurrent chemotherapy, and brachytherapy followed by two cycles of adjuvant cisplatin and fluorouracil and demonstrated that this treatment regimen was feasible. However, we used extended-field chemoradiation therapy, HDR brachytherapy, and four cycles of adjuvant carboplatin and paclitaxel chemotherapy for our patients; toxicity was acceptable and manageable. Verghese et al,^6^ in a phase II trial, used cisplatin and paclitaxel concurrent with radiotherapy in an attempt to improve the outcome for locally advanced cervical cancer in Indian women. The trial failed to show any improvement in response compared with cisplatin alone, and toxicity was greater in the combination-chemotherapy arm. At our center, we used single-agent cisplatin concurrent with radiotherapy followed by the combination of carboplatin and paclitaxel as adjuvant chemotherapy, and toxicity was less than grade 3 and manageable.

To date, no randomized trial is available to assess the impact of carboplatin and paclitaxel as adjuvant combination chemotherapy after chemoradiation therapy in locally advanced disease. A review by Tangjitgamol et al^1^ found evidence to use adjuvant chemotherapy after chemoradiation therapy for locally advanced cervical cancer. Recently, such a trial was initiated in Australia^7^ in which patients with International Federation of Gynecology and Obstetrics (FIGO) stages IB1 to IVA cervical cancer were randomly assigned to receive four cycles of adjuvant carboplatin and paclitaxel or to receive no additional chemotherapy after completion of chemoradiation therapy. However, we opted to offer this treatment to patients who had FIGO stage IVB disease (para-aortic lymphadenopathy). Patient case 2 had residual 4-cm cervical cancer at the vaginal vault, and she received salvage chemoradiation therapy to the pelvis and para-aortic nodal region. To the pelvic mass, she received a total of 63 Gy at 1.8 Gy/fraction after three-dimensional conformal radiotherapy. Also, she completed her 5 years of follow-up and is free of any recurrent disease. There is evidence of adequate local control with radical radiotherapy of residual disease after inadequate hysterectomy for cervical cancer.^8-10^

Petrić Miše et al^11^ and Jelavić et al^12^ reported encouraging results of four adjuvant cycles of cisplatin and ifosfamide chemotherapy after completion of concurrent chemoradiotherapy in an attempt to improve distant-relapse–free survival. In a randomized trial, Wong et al^13^ showed improvement in overall survival after administration of adjuvant chemotherapy with epirubicin at 90 mg/m^2^ compared with pelvic radiotherapy and no additional chemotherapy.

This is a unique report of treatment for patients with stage IVB disease (para-aortic lymphadenopathy). In the modern era of oncology, oncologists come across patients with advanced-stage disease not infrequently, and the treating oncologist, patients, and families often fail to understand the prognosis and goals of treatment, which can lead to a poor outcome of treatment.^14^ Should the FIGO stage IVB disease be segregated into two groups, patients with widespread distant metastases and those with para-aortic nodes only. Should the patients with para-aortic disease only have a better prognosis, provided they were treated aggressively. This question may get its answer in the near future. We hope that the results of the OUTBACK trial (ClinicalTrials.gov No. NCT01414608) will be able to resolve the question of whether to use adjuvant chemotherapy administration in locoregionally advanced cervical cancer.^7^
